# Extravascular lung water in critical care: recent advances and clinical applications

**DOI:** 10.1186/s13613-015-0081-9

**Published:** 2015-11-06

**Authors:** Mathieu Jozwiak, Jean-Louis Teboul, Xavier Monnet

**Affiliations:** Faculté de Médecine, Université Paris-Sud, Université Paris-Saclay, Le Kremlin Bicêtre, France; AP-HP, Service de réanimation médicale, Hôpital de Bicêtre, 78, rue du Général Leclerc, 94270 Le Kremlin-Bicêtre, France; Inserm UMR_S 999, Hôpital Marie Lannelongue, Le Plessis-Robinson, France

**Keywords:** Acute lung injury, Acute respiratory distress syndrome, Extravascular lung water, Fluid management, Fluid responsiveness, Hemodynamic monitoring, Lung oedema, Pulmonary vascular permeability index, Transpulmonary thermodilution

## Abstract

Extravascular lung water (EVLW) is the amount of fluid that is accumulated in the interstitial and alveolar spaces. In lung oedema, EVLW increases either because of increased lung permeability or because of increased hydrostatic pressure in the pulmonary capillaries, or both. Increased EVLW is always potentially life-threatening, mainly because it impairs gas exchange and reduces lung compliance. The only technique that provides an easy measurement of EVLW at the bedside is transpulmonary thermodilution. The validation of EVLW measurements by thermodilution was based on studies showing reasonable correlations with gravimetry or thermo-dye dilution in experimental and clinical studies. EVLW should be indexed to predicted body weight. This indexation reduces the proportion of ARDS patients for whom EVLW is in the normal range. Compared to non-indexed EVLW, indexed EVLW (EVLWI) is better correlated with the lung injury score and the oxygenation and it is a better predictor of mortality of patients with acute lung injury or acute respiratory distress syndrome (ARDS). Transpulmonary thermodilution also provides the pulmonary vascular permeability index (PVPI), which is an indirect reflection of the integrity of the alveolocapillary barrier. As clinical applications, EVLWI and PVPI may be useful to guide fluid management of patients at risk of fluid overload, as during septic shock and ARDS. High EVLWI and PVPI values predict mortality in several categories of critically ill patients, especially during ARDS. Thus, fluid administration should be limited when EVLWI is already high. Whatever the value of EVLWI, PVPI may indicate that fluid administration is particularly at risk of aggravating lung oedema. In the acute phase of haemodynamic resuscitation during septic shock and ARDS, high EVLWI and PVPI values may warn of the risk of fluid overload and prevent excessive volume expansion. At the post-resuscitation phase, they may prompt initiation of fluid removal thereby achieving a negative fluid balance.

## Introduction

Extravascular lung water (EVLW) is the amount of water that is contained in the lungs outside the pulmonary vasculature. It corresponds to the sum of interstitial, intracellular, alveolar and lymphatic fluid, not including pleural effusions [1]. An increase in EVLW is the pathophysiological hallmark of hydrostatic pulmonary oedema and acute respiratory distress syndrome (ARDS) [2]. EVLW is also high in many septic shock [3] and critically ill [4] patients. For many years, this variable of paramount importance in the pathophysiology of critical illness could only be measured ex vivo. The emergence of transpulmonary thermodilution has opened up the area of EVLW investigation in the clinical setting.

During recent years, many studies have been dedicated to EVLW in the field of critical care and ARDS research. They have focused on the validation of its measurement and on its value for the characterisation of lung oedema, for the prognostic stratification of critically ill patients, for the evaluation of lung-targeted treatments and for the strategy of fluid management.

We have sought to provide a comprehensive review of these recent advances. We also attempted to consider the different clinical applications in which EVLW could help manage critically ill patients.

## The physiology of lung water

**Fig. 1:**
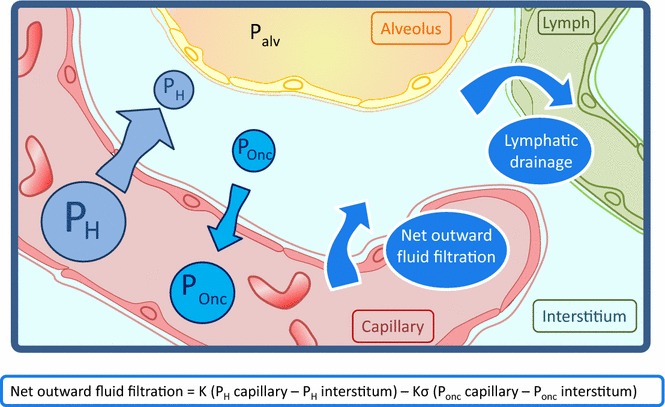
Physiology of lung water. There is a physiological net outward fluid filtration from microvessels to the interstitium governed by the Starling’s law, which is strictly controlled by the lymphatic drainage system (*P*_alv_ alveolar pressure, *P*_H_ hydrostatic pressure, *P*_onc_ oncotic pressure, *K* filtration coefficient of the alveolocapillary barrier, *K*σ reflection coefficient of the alveolocapillary barrier)

Physiologically, there is a normal leakage of fluid and solutes from the pulmonary microvessels into the pulmonary interstitial tissue. Fluid and solutes do not reach the alveoli because of the tight junctions of the alveolar epithelium. This net outward fluid filtration from microvessels to the interstitium is governed by Starling’s law, which mainly includes the gradient of hydrostatic and oncotic pressures between the vascular and interstitial spaces and the filtration coefficient of the alveolocapillary barrier [5–8] (Fig. 1).

To preserve their function of oxygenation, the lungs must be kept dry [5]. The volume of EVLW is strictly controlled by the lymphatic drainage system, which constantly removes EVLW from the interstitial tissue and pours it into the superior vena cava through the thoracic duct. In the normal lung, the value of the normal EVLW indexed to the body weight (EVLWI), which results from the equilibrium between fluid leakage and lymphatic drainage, is <7 mL/kg of body weight [9]. In a series of 534 normal lungs, Tagami and colleagues reported a value of EVLWI of 7.3 ± 2.8 mL/kg [10], suggesting that normal values of EVLWI may be <10 mL/kg.

**Fig. 2:**
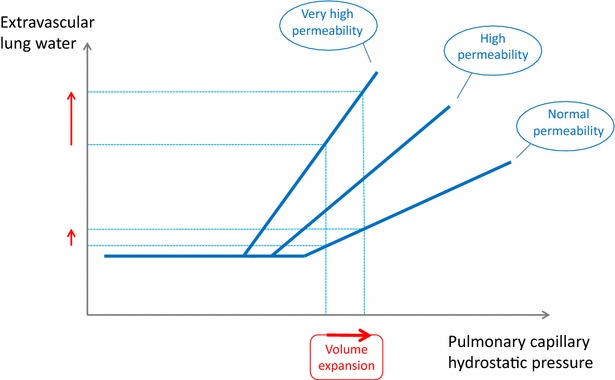
Relationship between extravascular lung water and pulmonary capillary hydrostatic pressure for different levels of pulmonary vascular permeability. The higher the lung permeability, the greater the risk of increase in extravascular lung water during volume expansion

Increased interstitial EVLW can occur as the result of increased pulmonary microvascular hydrostatic pressure or of decreased blood oncotic pressure or as the result of increased permeability of the alveolocapillary barrier, as typically in ARDS. In ARDS, the larger the increase in pulmonary microvascular permeability, the greater the outward fluid filtration from microvessels, at any given pulmonary microvascular hydrostatic pressure [7]. Also, during ARDS, if the pulmonary microvascular hydrostatic pressure increases, frequently occuring as the result of fluid resuscitation in associated circulatory failure, the increase in EVLW is all the more pronounced as impairment of pulmonary permeability increases (Fig. 2).

At the early stages, the increase in interstitial EVLW is regulated by the compensatory increase of lymphatic drainage. However, at high fluid filtration rate, lymphatic drainage is overwhelmed and interstitial EVLW enters the alveoli [6–8]. In hydrostatic pulmonary oedema, another mechanism also contributes to the protection of the lung against fluid overload: excess fluid is removed from the alveoli to the interstitial tissue across the alveolar epithelial barrier by active ion transport [11, 12]. In ARDS, this alveolar fluid clearance is impaired and the lymphatic network is injured, all of which contributing to the accumulation of EVLW.

## How to measure EVLW?

In clinical practice, the positive diagnosis of pulmonary oedema is based on clinical examination and chest X-ray. Nevertheless, quantifying the volume of EVLW from clinical examination and chest X-ray is much more complex, because of interobserver variability and the lack of sensitivity of this approach [13, 14]. Other methods are thus required to measure EVLW directly. The gold standard is gravimetry [15]. This ex vivo method consists in measuring the difference in the weight of the lungs before and after they have been dried out. Of course, this method cannot be used in living patients.

EVLW can be estimated by computed tomography [16] or magnetic resonance imaging [15], but such techniques are not appropriate for convenient and repeated assessment. Isotopic methods [17] can only be used for research and electrical impedance tomography [18] is currently not sufficiently validated.

Lung ultrasonography is increasingly used to detect lung oedema [19]. Nevertheless, its ability to quantify the volume of EVLW is not established. There is no defined method for grading the severity of typical ultrasonography signs of lung oedema. Moreover, ultrasonography may be limited by the fact that it assesses lung oedema in some specific regions and not in the whole organ.

**Fig. 3:**
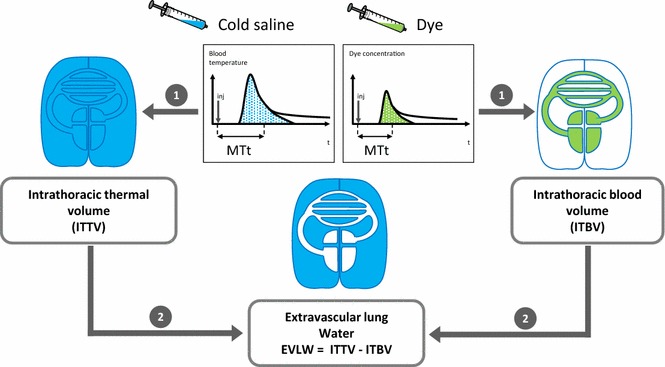
Measurement of extravascular lung water by thermo-dye dilution (*MTt* mean transit time)

As a first clinical alternative, transpulmonary thermo-dye dilution, which can be considered as the in vivo gold standard for EVLWI measurement, has been developed and validated versus gravimetry in animals [20] and human beings [21]. The technique requires a central venous catheter inserted in the superior vena cava territory and a thermistor-tipped arterial catheter placed in the femoral artery. The thermo-dye dilution is performed by injecting simultaneously through the central venous catheter a cold indicator (cold saline) and a colorimetric indicator (indocyanine green). The volume of distribution of the cold indicator includes the intravascular and the extravascular spaces of the intrathoracic compartment, whereas the colorimetric indicator is strictly an intravascular indicator. Thus, the measurement of EVLWI is obtained by subtracting the volume of distribution of these two indicators (Fig. 3). Nevertheless, this method is cumbersome and costly and has been advantageously replaced by the transpulmonary thermodilution technique.

### Principles of transpulmonary thermodilution

With this technique, the patient must have a central venous catheter in the superior vena cava territory and a thermistor-tipped arterial catheter, most often in the femoral artery. It can also be inserted in the axillary, brachial and radial arteries [22]. The thermodilution measurement is performed by injecting cold saline through the central venous catheter. The ensuing decrease in blood temperature is detected by the thermistor-tipped arterial catheter, thus yielding a thermodilution curve.

**Fig. 4:**
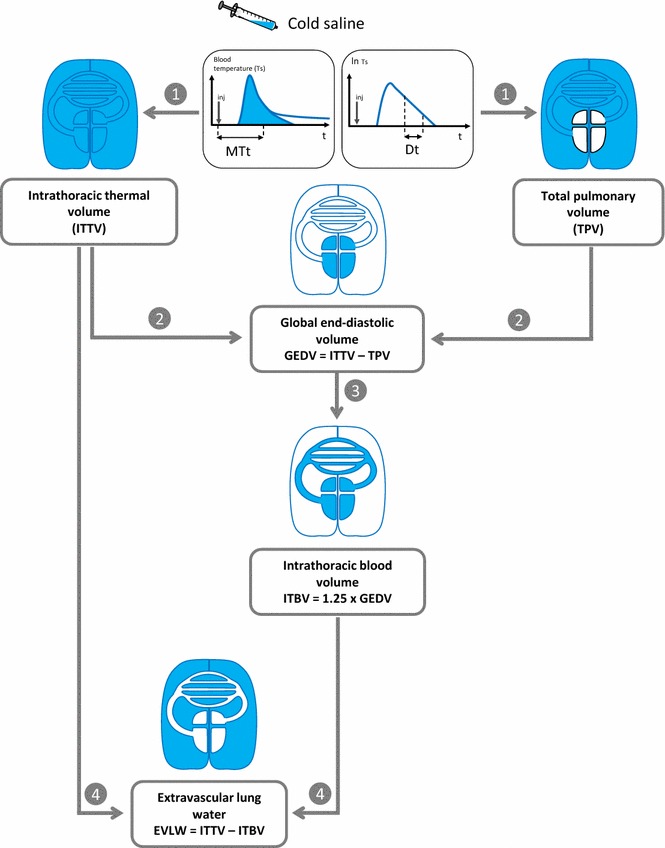
Measurement of extravascular lung water by single thermal indicator dilution (*MTt* mean transit time, *Dt* downslope time)

EVLWI is estimated from an analysis of the thermodilution curve that is based on both the Stewart–Hamilton and Newman principles [23, 24] (Fig. 4). According to the Stewart–Hamilton principle, the total distribution volume of the cold indicator between the injection and the detection sites is obtained by multiplying cardiac output by the mean transit time of the cold indicator provided by the thermodilution curve: intrathoracic thermal volume (ITTV) = cardiac output × mean transit time (Fig. 4). According to the Newman principle, the largest distribution volume of the cold indicator between the injection and the detection sites, which is the total pulmonary volume, is obtained by multiplying cardiac output by the downslope time of the thermodilution curve (Fig. 4).

The measurement of EVLWI then requires two more steps. First, the global end-diastolic volume (GEDV), which is the sum of the maximal volumes of the four cardiac chambers, is obtained by subtracting the total pulmonary volume from ITTV (Fig. 4). Second, the intrathoracic blood volume (ITBV) is estimated from the GEDV according to the equation: ITBV = GEDV × 1.25 [25, 26] (Fig. 4). The latter estimation is inferred from a study in which ITBV was measured by thermo-dye and single thermodilution [25]. From the thermo-dye dilution measurements, the authors showed that ITBV and GEDV were linearly correlated and that ITBV = (1.25 × GEDV) − 28.4 mL. In a validation population, the ITBV estimated through this equation from GEDV measured by single thermodilution was a reliable estimation of the ITBV measured by thermo-dye thermodilution [25]. This relationship between ITBV and GEDV has been confirmed in cardiac surgical patients [26]. Finally, EVLWI is obtained by subtracting ITBV from ITTV (Fig. 4).

### Validation of EVLW estimated by transpulmonary thermodilution

The validity of the measurement of EVLWI by transpulmonary thermodilution has been established and consolidated in recent years. EVLWI measured by transpulmonary thermodilution was reasonably correlated with the value measured by thermo-dye dilution in humans [25, 27] and with the value obtained through gravimetry in animals [28–30]. More recently, the validation in human beings came from an autopsy study in which the value of EVLWI measured by transpulmonary thermodilution before death well correlated with the value obtained by gravimetry after autopsy [9].

The reliability of EVLWI estimated by transpulmonary thermodilution was also suggested by studies showing the good precision of the measurement in animals [31, 32] and in patients [33, 34]. For instance, Dres and colleagues reported that transpulmonary thermodilution was able to detect the small and short-term changes in EVLWI induced by bronchoalveolar lavage [34]. The increase induced by the lavage that was detected by thermodilution was very close to the volume of saline that could not be removed by suction during the procedure and was left in the lungs.

Finally, another argument in favour of the validity of thermodilution for estimating EVLWI comes from studies showing that EVLWI predicts mortality in critically ill, septic and ARDS patients independently of other severity indices [35–38]. This indirectly validates the estimation of EVLWI by transpulmonary thermodilution since such a prediction of mortality would be impossible to demonstrate if the measurement of EVLWI were not valid. In this study, the fact that the result could be influenced by the mathematical coupling between EVLWI and biometric data (weight and height) that themselves influence prognosis [39] was avoided by the inclusion of these biometric data in the multivariate analysis model.

### The issue of EVLW indexation

Historically, EVLW measurement has been indexed to the weight of patients at the time of the measurement [40]. However, the main determinant of lung volume is not the patient’s weight, but rather the patient’s height, and perhaps the gender [41]. Two recent studies have suggested that EVLW should be indexed to height only [42, 43]. Height was the only biometric parameter independently associated with EVLW measurements [42]. Indexing EVLW to the weight underestimates EVLW in the case of overweight, a condition that is common in critically ill patients due to the positive fluid balance. In this regard, it has been observed that indexing EVLW to predicted body weight reduced the proportion of ARDS patients for whom EVLWI was in the normal range [40]. EVLWI was better correlated with lung injury score and oxygenation [40, 44] and was a better predictor of mortality in acute lung injury (ALI)/ARDS patients if indexed to predicted rather than to actual body weight [44, 45].

### Practical aspects of EVLW measurements

The values of EVLWI provided by three successive transpulmonary thermodilution measurements should be averaged. When three cold boluses are used, the least significant change in EVLWI is 12 % [33]. Roughly, this means that changes in EVLWI of more than one unit can be considered as significant.

Although injecting the cold bolus in the superior vena cava territory is the common method for transpulmonary thermodilution, the femoral vein could also be used [46]. Nevertheless, injection in the superior vena cava territory should be considered as the validated reference. It has been suggested that room temperature boluses could be used instead of iced ones, without changing the reliability of transpulmonary thermodilution [47]. Nevertheless, a recent study suggested that room temperature boluses result in slight but significant overestimation of EVLWI [48].

### Commercially available devices

The two transpulmonary thermodilution devices that are currently commercially available (PICCO2, Maquet^®^, Munich, Germany and VolumeView/EV 1000^®^, Edwards Lifesciences, Irvine, CA, USA) both use the Stewart–Hamilton principle to calculate the cardiac output and the same algorithm to calculate EVLWI, even if they use a different algorithm to assess GEDV [49, 50].

Whereas the PICCO monitor assesses GEDV according to the Newman principle, the VolumeView/EV 1000 monitor assesses it from the maximum up-slope and the maximum down-slope of the thermodilution curve and a proprietary function [50]. An experimental study has shown that under different haemodynamic and respiratory conditions (hypovolaemia, hypervolaemia, inotropic stimulation, ARDS), the EVLWI measurements provided by both devices were comparable [49]. A more recent human study has confirmed that EVLWI values provided by these two devices are interchangeable [50].

## Limitations of EVLW measurement by transpulmonary thermodilution

The limitations of EVLWI measurement by transpulmonary thermodilution and the conditions in which it is not reliable have been better and better characterised (Table 1).

**Table 1:** Limitations of extravascular lung water measurement by transpulmonary thermodilution

*Overestimation of extravascular lung water*	
Positive end-expiratory pressure (potential)	
Lung resection (proven)	
*Underestimation of extravascular lung water*	
Positive end-expiratory pressure (potential)	
Pulmonary vascular occlusion (proven)	
Heterogeneous lung injury (potential)	
Pleural effusions (plausible)	

### Pulmonary vascular occlusion

The accuracy of EVLWI measurement depends on the volume of distribution of the cold indicator, i.e. the ITTV. This means that transpulmonary thermodilution can only detect EVLW in lung regions that are reached by the cold indicator, i.e. in well-perfused areas.

In experimental studies, the occlusion of large pulmonary arteries led to underestimation of EVLWI by transpulmonary thermo-dye dilution compared with gravimetry, which was corrected after the reopening of the arteries [51–53]. However, underestimation of EVLWI was observed only when large vessels were occluded [51, 53], not small vessels [52]. The clinical implication is that the value of EVLWI measured by thermodilution is likely underestimated in the case of pulmonary embolism. During ARDS, the occlusions of the pulmonary vasculature that could result from vascular remodelling, microthrombi, hypoxic vasoconstriction or positive end-expiratory pressure (PEEP) [54] mainly affect the small vessels. This suggests that the accuracy of EVLWI measurement should not be impaired by vascular occlusion in this clinical instance.

### Lung resection

Logically, lung resection decreases the volume of EVLW. Two experimental studies [55, 56] showed that EVLWI measurement by transpulmonary thermo-dye dilution or thermodilution decreased after pneumonectomy, but was correlated with the gravimetric EVLWI measurement. Nevertheless, the absolute value of EVLWI measured by transpulmonary thermodilution was overestimated compared with the EVLWI measurement by gravimetry [55]. Similarly, a recent experimental study demonstrated that EVLWI measurement by transpulmonary thermodilution was significantly affected by 1-lung ventilation [57]. Thus, in ARDS patients with pneumonectomy or 1-lung ventilation, EVLWI measurement by transpulmonary thermodilution should be interpreted with caution, though the ability of the technique to measure changes in EVLWI should be unaffected.

### Type of ARDS

Experimental studies have shown that EVLWI measurement by transpulmonary thermodilution is correlated with the gravimetric measurement in a homogeneous lung injury indirectly induced by oleic acid, but is underestimated in a heterogeneous lung injury directly induced by hydrochloric acid [58–60]. This underestimation of EVLWI was attributed to a redistribution of the pulmonary blood flow away from the oedematous areas.

Nevertheless, using PET scan, Schuster and colleagues demonstrated that in ALI/ARDS patients there was no difference in regional pulmonary perfusion pattern with healthy subjects and that mechanisms such as hypoxic vasoconstriction allowing redistribution of pulmonary blood flow away from oedematous lung regions were severely blunted [61]. Therefore, it can be supposed that the type of ALI/ARDS would not alter the accuracy of EVLWI measurement by transpulmonary thermodilution.

### PEEP

In theory, the effects of PEEP on EVLWI measurement are complex and result from opposite mechanisms. First, PEEP could alter the reliability of transpulmonary thermodilution by having opposite effects on the diffusion volume of the cold indicator. On the one hand, high levels of PEEP could reduce the cold indicator diffusion volume by squeezing pulmonary microvessels, leading to an underestimation of EVLWI [62]. On the other hand, high levels of PEEP could increase the cold indicator diffusion volume by recruiting some atelectatic lung regions and reducing hypoxic vasoconstriction, leading to an overestimation of EVLWI [58].

Second, PEEP could actually change the volume of EVLWI through, here again, opposite mechanisms. By decreasing cardiac output, PEEP may reduce the pulmonary microvascular hydrostatic pressure and hence EVLWI [63]. Also, during ARDS, by increasing the central venous pressure, PEEP could impede the lymphatic drainage of EVLWI [64]. By contrast, during hydrostatic pulmonary oedema, PEEP could contribute to the decrease in EVLWI by improving cardiac function through its own haemodynamic effects [65].

The net consequence of these theoretical mechanisms in clinical practice is unclear. In a study conducted in ARDS patients, there was a strong correlation between the EVLWI measured by transpulmonary thermo-dye dilution and the lung weight measured by computed tomography in a broad range of PEEP levels (from 10 to 20 cmH_2_O) [16]. This may suggest that the effects of PEEP on EVLWI measured by transpulmonary thermodilution are mild or negligible. Nevertheless, we lack clinical studies in which all the determinants of EVLW formation and drainage are investigated at various levels of PEEP.

### Pleural effusions

For many years, it has been claimed that EVLWI will be overestimated in the case of pleural effusions because the pleural fluid might contribute to the dilution of the cold bolus injected in the superior vena cava. Nevertheless, this is unlikely because of the distance between fluid in the pleural cavity and the pulmonary vasculature. Accordingly, an experimental study previously showed that pleural effusion in dogs had no effect on the values of EVLWI measured by double-indicator dilution technique [66]. Moreover, a recent clinical study reported that large-volume thoracentesis resulted not in a decrease, but in an increase in transpulmonary thermodilution-derived EVLWI [67]. This increase in EVLWI was likely explained by the expansion of some atelectatic regions with thoracocentesis or, less likely, by the occurrence of a post-thoracentesis hydrostatic pulmonary oedema. Some other studies are needed to confirm these former results.

### Other potential limitations

Renal replacement therapy could in theory affect the reliability of transpulmonary thermodilution by inducing leakage of the cold indicator between the injection site and the arterial thermometer. However, the flow of the extracorporeal circuit is likely not enough to be significant. This has been clearly shown by two studies [68, 69], including one in which the flow rate of blood pump during continuous veno-venous hemofiltration was as high as 300 mL/min [69].

Of course, transpulmonary thermodilution is not reliable under extracorporeal membrane oxygenation. Therapeutic hypothermia likely does not affect the drift in blood temperature induced by cold bolus injection, what explains why transpulmonary thermodilution measurement of EVLWI is reliable in this situation [70].

The complications inherent to transpulmonary thermodilution technique are in fact related to the venous and arterial catheterisations [71]. In particular, the arterial catheter is of larger diameter than common arterial cannula, because it includes a thermistor. Nevertheless, in a multicentre review of 514 catheters, the incidence of limb ischemia and femoral artery thrombosis was rare (0.4 and 0.2 %, respectively) [71]. Although the radial and axillary arteries could be used for arterial cannulations with specific models of catheters, these routes are less convenient than the femoral one.

Finally, the thermistor-tipped arterial catheter required for transpulmonary thermodilution costs around 200 euros. No cost–benefit study has already been performed in this domain.

## The pulmonary vascular permeability index

In addition to EVLWI, transpulmonary thermodilution is a unique method for estimating the permeability of the pulmonary capillary barrier through calculation of the PVPI. It is the ratio between EVLWI and pulmonary blood volume [2, 72, 73], i.e. the ratio between the volume of fluid that has leaked toward the extravascular spaces and the volume of fluid that has remained in the intravascular compartment. The PVPI is automatically provided by the transpulmonary thermodilution system each time a cold bolus is injected.

It is of importance to note that the PVPI automatically displayed by the PiCCO device is reliable only when the central venous catheter is inserted in the superior vena cava territory [74]. Whether the central venous catheter is inserted in the femoral vein, the PVPI displayed will be underestimated [74]. Indeed, PVPI is indirectly calculated from the GEDVI, which is overestimated in case of femoral venous access due to the additional volume of vena cava inferior participating in transpulmonary thermodilution [74]. While the PiCCO device automatically corrects GEDVI in the case of femoral venous access, this correction is not yet used for the calculation of PVPI [74].

The value of PVPI has been validated by some animal [28] and human [2, 72, 73] studies showing that it was significantly higher in patients with ALI/ARDS than in patients with hydrostatic pulmonary oedema. In a series of patients with pulmonary oedema, a PVPI value of 3 was the best threshold to distinguish between both forms of pulmonary oedema [2, 72] and should thus be considered as the maximal normal value. Although PVPI is an indirect estimation of lung permeability, it is the only way to evidence an injury of the alveolocapillary barrier and to quantify the pulmonary leak at the bedside.

## The prognostic value of EVLW and PVPI

EVLWI and PVPI have been shown to predict mortality in diverse categories of critically ill patients (Table 2). EVLWI predicted mortality in severe sepsis or septic shock [3, 73, 75, 76], but also in burned patients [77] and in a general population of critically ill patients [4]. In some of these studies, EVLWI was shown to predict mortality independently of other markers of disease [75, 78]. Some of these studies were included in a meta-analysis, which confirmed this prognostic value of EVLWI in critically ill patients [79]. In the context of severe sepsis or septic shock, PVPI has also been shown to be significantly higher in non-survivors than in survivors [73, 78].

**Table 2:** Prognostic value of extravascular lung water in critically ill patients

	Study	Number of patients	Type of EVLW indexation	Prognostic value
*General critically ill patients*	Sakka et al. [4]	373	Actual body weight	Independent predictor of ICU mortality
*Severe sepsis or septic shock patients*	Martin et al. [3]	29	Actual body weight	Higher EVLWI in ICU non-survivors
	Chung et al. [75]	33	Actual body weight	Independent predictor of in-hospital survival
	Chung et al. [76]	67	Actual body weight	Independent factor for the development of MODS
	Chew et al. [73]	51	Actual and predicted body weight	Higher EVLWI in ICU non-survivors
	Mallat et al. [78]	55	Actual and predicted body weight	Independent predictor of ICU mortality
*ARDS patients*	Philips [85]	59	Actual and predicted body weight	Good predictor of ICU mortality
	Craig et al. [45]	44	Predicted body weight	Independent predictor of ICU mortality
	Brown et al. [37]	59	Predicted body weight	Independent predictor of ICU mortality
	Jozwiak et al. [36]	200	Predicted body weight	Independent predictor of Day-28 mortality

*ARDS:* acute respiratory distress syndrome, *EVLWI:* indexed extravascular lung water, *ICU:* intensive care unit, *MODS:* multiple organ dysfunction syndrome

In the specific clinical setting of ARDS, it has also been repeatedly observed that high values of EVLWI are significantly associated with mortality [35–37, 44, 45, 80]. Decrease in EVLWI during the first 48 h of ARDS may be associated with 28-day survival [38]. EVLWI has been shown to be a good predictor of mortality [36, 37, 44, 45]. In 200 ARDS patients, our group reported that the maximum value of EVLWI recorded during the course of ARDS, but not the value of EVLWI at day-1 predicted day-28 mortality in an independent manner [36]. It is noteworthy that the maximum value of EVLWI was reached within 3 days on average [36].

It is interesting to note that EVLWI and indices of oxygenation have both been reported to be independent predictors of ARDS [36]. This suggests that both markers have their own physiological meaning. Poor oxygenation in ARDS results not only from EVLW accumulated in the alveoli and interstitium, but also from some other abnormalities such as atelectasis or arteriovenous shunts due to pulmonary vascular injury [54]. This suggests that both oxygenation indices and EVLWI should be evaluated to assess the severity of ARDS.

PVPI was also found to be related to the prognosis of ARDS patients [36, 80] and to predict mortality in an independent manner [36]. Both PVPI and EVLWI predict mortality in an independent way, which again suggests that they indicate a different pathophysiological pattern of ARDS. While PVPI appears to characterise the degree of impairment of the alveolocapillary barrier itself, EVLWI seems to indicate the severity of the pulmonary leak resulting from this injury.

## How to use EVLW and PVPI in clinical practice?

### Definition of ARDS

Neither EVLWI nor PVPI measurements have been included in the new Berlin definition of ARDS [81], due to concerns of availability [82]. However, several authors have suggested that, as pathophysiological hallmarks of ARDS, EVLWI and/or PVPI should be taken into account when defining the disease [83–85]. EVLWI and PVPI may potentially improve the definition of ARDS, as is suggested by at least three arguments.

First, an increase in pulmonary vascular permeability is fundamentally the functional hallmark of ARDS. In the Berlin definition of ARDS, impaired permeability is defined by the absence of high left ventricular filling pressure. However, this criterion is very indirect. Moreover, an increased left ventricular preload cannot exclude ARDS, since authentic ARDS may be accompanied by high filling pressure of the left ventricle, especially if the patient has been already resuscitated. In this regard, some clinical studies report that the left ventricular preload is actually high in not less than one-third of ARDS patients [86].

Second, it was recently found that among all patients who met the Berlin definition of ARDS, only 45 % had diffuse alveolar damage [87], while this is the pathological characteristic of ARDS. By contrast, in another study, an increase of EVLWI above 15 mL/kg identified patients with diffuse alveolar damage with 99 % certainty [10].

Third, some clinical studies directly suggest that taking EVLWI and/or PVPI values into account may help define ARDS and predict its prognosis. For instance, EVLWI predicted the progression to ALI in patients with risk factors 2.6 ± 0.3 days before the patients met American-European Consensus Conference criteria of ARDS [88]. In another study, the value of EVLWI was in close relationship with the severity of ARDS as defined by the categories of the Berlin definition [89]. It has also been shown that the use of EVLWI improves up to eightfold the post-test odds ratio for the diagnosis of ALI, ARDS and severe lung injury [73]. Even though these arguments plead in favour of the inclusion of EVLWI in the definition of ARDS, its value should be investigated by large-scale studies.

### Fluid management

It is today acknowledged that excessive fluid loading is associated with a higher risk of dying in several categories of patients. The cumulative fluid balance is an independent predictor of mortality in patients with septic shock [90], ARDS [36, 91] and acute kidney injury [92]. Likewise, a restrictive fluid strategy significantly reduces the duration of ventilation of ARDS patients [93]. In a retrospective series of ARDS patients, a negative fluid balance was found to be associated with decreased EVLWI during the first week after admission to the intensive care unit (ICU) and with a decrease of day-28 mortality [35]. The consistent results emerging from these studies are explained by the deleterious pleiotropic effects of tissue swelling [94], the most important being worsening of lung oedema.

However, fluid administration is the most widely used first-line treatment in acute circulatory failure. It is expected to increase cardiac preload and, in the case of preload dependence, to increase cardiac output and eventually improve tissue oxygenation [95]. Thus, every day in the ICU, clinicians have to face this therapeutic dilemma: for this patient with circulatory failure and lung impairment, should I opt for fluid administration?

Two considerations may help answer this question at the bedside. First, indices that have been developed to predict fluid responsiveness/unresponsiveness may be very useful [96]. If negative, they identify cases where fluid administration has no chance of having any haemodynamic benefits and should definitely be avoided. Second, EVLWI and PVPI may be used as criteria indicating the risk of fluid administration. A high EVLWI value indicates that lung oedema is already present and should obviously not be worsened. Whatever the level of EVLWI, an increased PVPI indicates the presence of a pulmonary leak and that any further fluid administration is particularly at risk of increasing EVLWI (Fig. 2). During the acute phase of resuscitation, high EVLWI and PVPI may serve as indicators for fluid restriction and encourage clinicians to choose alternative interventions for haemodynamic resuscitation. After initial haemodynamic stabilisation, during the de-escalation phase, EVLWI may be helpful in instituting an aggressive but controlled fluid removal strategy.

Supporting this, in the context of ARDS, some small studies suggest that management based on protocols including EVLWI measurements is safe [97], leads to a lower cumulative fluid balance [98], improves ICU mortality [97], and reduces the duration of mechanical ventilation [98] and of ICU stay [98, 99]. Nevertheless, a pivotal randomised trial comparing haemodynamic and fluid based on the values of EVLWI versus non-EVLWI-guided therapy in ARDS or septic patients is necessary to confirm the results of these small studies. In this regard, an ongoing large-scale prospective study in ARDS is comparing a fluid management therapy based on central venous pressure and urine output with a strategy based on the values of EVLWI and indicators of preload reserve (HEAL Study, NTC00624650).

The value of guiding the therapeutic strategy by measuring EVLWI has been investigated in clinical settings other than ARDS. After subarachnoid haemorrhage managed by transpulmonary thermodilution with an algorithm including EVLWI, patients had a lower rate of vasospasm and cardiopulmonary complications compared with those managed with standard therapy [100]. In patients undergoing cardiac surgery, therapeutic algorithms including EVLWI in addition to other transpulmonary thermodilution indices also yield some clinical benefits [101, 102]. EVLWI was also found to be useful in guiding fluid removal in patients with renal replacement therapy [103].

### Weaning from mechanical ventilation

One of the main causes of weaning failure is cardiac failure induced by the transfer from positive to negative pressure ventilation [104]. For decades, the diagnosis of weaning-induced pulmonary oedema has been based on the measurement of pulmonary artery occlusion pressure with the pulmonary artery catheter [105], which nowadays is much less used than in the past [106].

Recently, our group investigated the ability of EVLWI measured by transpulmonary thermodilution to evidence pulmonary oedema in this specific context. We showed that an increase in EVLWI by more than 14 % between the beginning and end of a T-tube weaning trial was able to diagnose weaning-induced pulmonary oedema with a sensitivity of 67 % and a specificity of 100 % [107]. In our opinion, these results should not encourage the insertion of a transpulmonary thermodilution device for the sole purpose of evidencing cardiac dysfunction in a difficult-to-wean patient, since less costly and less invasive methods are available [108]. Rather, we suggest that, if a transpulmonary thermodilution device is still in place at the time of weaning from mechanical ventilation, attention should be paid to the changes in EVLWI during the spontaneous breathing trials.

### Other potential clinical situations

In lung transplantation, the major criterion currently used to evaluate the viability of pulmonary grafts before transplantation is the ratio between partial pressure of arterial oxygen and the fraction of inspired oxygen [109]. Nevertheless, this criterion might be insufficient for adequate assessment of the suitability of lungs for transplantation [109]. Three recent studies [110–112] have shown that EVLWI measured by transpulmonary thermodilution could be a useful parameter in this context. It is able to assess pulmonary oedema during ex vivo lung perfusion [112] and in potential lung donors [111]. Moreover, it predicts the suitability of the pulmonary grafts for transplantation [110, 111].

EVLWI and PVPI may also be used to assess the effects of some specific therapeutic interventions during ARDS. For instance, the effects of prone positioning on EVLWI have been investigated. While one study reported that EVLWI does not change to a relevant extent 10 min [113] or up to 6 h after prone positioning [114], another study found that it significantly decreased after 18 h in prone position [115]. This suggests that EVLWI may follow the improvement of the other markers of pulmonary function during the postural manoeuvre, even though these observations need to be confirmed. Data are lacking to explain the pathophysiological mechanisms underlying these potential changes. For instance, the degree of association between lung recruitment induced by prone positioning and the level of EVLWI has not been investigated.

## Conclusions

Transpulmonary thermodilution has emerged as the technique allowing clinicians to estimate the volume of lung oedema at the bedside. PVPI, which is derived from the EVLWI measurement, is an indirect marker of the permeability of the alveolocapillary barrier. In spite of some limitations, measurements of EVLWI and PVPI should now be considered as accurate and precise. As clinical applications, measuring EVLWI quantifies pulmonary oedema and PVPI distinguishes between hydrostatic and permeability lung oedema. A large-scale study should confirm the utility of including EVLW and PVPI in the common management of ARDS. EVLWI and PVPI may contribute to a better definition of ARDS, even though this still needs to be. EVLWI and PVPI may mostly be used to conduct fluid resuscitation in a safe and controlled manner, especially in septic shock combined with ARDS. High EVLWI and PVPI values may indicate that further fluid administration risks fluid overload and, at the post-acute phase, that fluid removal should be initiated.

## Abbreviations

ALI: acute lung injury
ARDS: acute respiratory distress syndrome
FiO_2_: inspired oxygen fraction
EVLW: extravascular lung water
GEDV: global end-diastolic volume
ICU: intensive care unit
ITBV: intrathoracic blood volume
ITTV: intrathoracic thermal volume
PaO_2_: arterial oxygen tension
PVPI: pulmonary vascular permeability index
PEEP: positive end-expiratory pressure
